# Stem Cell Plasticity and Dormancy in the Development of Cancer Therapy Resistance

**DOI:** 10.3389/fonc.2019.00626

**Published:** 2019-07-10

**Authors:** Maria Laura De Angelis, Federica Francescangeli, Filippo La Torre, Ann Zeuner

**Affiliations:** ^1^Department of Oncology and Molecular Medicine, Istituto Superiore di Sanità, Rome, Italy; ^2^Department of Surgical Sciences Policlinico Umberto I, Sapienza University of Rome, Rome, Italy

**Keywords:** cancer stem cells, chemoresistance, dormancy, quiescence, plasticity, drug resistance, target therapies

## Abstract

Cancer treatment with either standard chemotherapy or targeted agents often results in the emergence of drug-refractory cell populations, ultimately leading to therapy failure. The biological features of drug resistant cells are largely overlapping with those of cancer stem cells and include heterogeneity, plasticity, self-renewal ability, and tumor-initiating capacity. Moreover, drug resistance is usually characterized by a suppression of proliferation that can manifest as quiescence, dormancy, senescence, or proliferative slowdown. Alterations in key cellular pathways such as autophagy, unfolded protein response or redox signaling, as well as metabolic adaptations also contribute to the establishment of drug resistance, thus representing attractive therapeutic targets. Moreover, a complex interplay of drug resistant cells with the micro/macroenvironment and with the immune system plays a key role in dictating and maintaining the resistant phenotype. Recent studies have challenged traditional views of cancer drug resistance providing innovative perspectives, establishing new connections between drug resistant cells and their environment and indicating unexpected therapeutic strategies. In this review we discuss recent advancements in understanding the mechanisms underlying drug resistance and we report novel targeting agents able to overcome the drug resistant status, with particular focus on strategies directed against dormant cells. Research on drug resistant cancer cells will take us one step forward toward the development of novel treatment approaches and the improvement of relapse-free survival in solid and hematological cancer patients.

## Introduction

Resistance to chemotherapy and molecularly targeted therapies is a major problem that limits the effectiveness of cancer treatments. While some tumors are intrinsically insensitive to therapies due to pre-existing resistance factors (primary or intrinsic resistance), others become resistant during drug treatment (1). The development of resistance after an initial period of response (acquired resistance) is due to the molecular heterogeneity of tumor cells which, together with their ability to evolve at the genetic, epigenetic, and phenotypic level, is able to overcome the action of cancer therapies. The emergence of resistant cells has been observed upon treatment with chemotherapy, radiotherapy, and targeted therapies, including EGFR tyrosine kinase inhibitors in lung cancer, anti-HER2 therapies in breast cancer, and BRAF inhibitors in melanoma. Even cancer immunotherapies, which exploit a dynamic interaction between the host immune system and tumor cells thus achieving lasting antitumor responses, are linked to the development of resistance and consequent cancer progression (2). Treatment with chemotherapeutic or targeted drugs is increasingly recognized to promote the emergence of resistant cells with features of cancer stem cells (CSCs) (3). This process clearly involves a Darwinian selection of cell populations with novel genetic mutations conferring drug resistance (4–6). However, non-genetic events involving both chromatin remodeling and the activation of stress-related pathways are responsible for the establishment of drug tolerance, a process more rapid, and massive than genetic mutation (7–9). Drug tolerance is habitually associated to a transient state of slow proliferation, thus identifying a population of Drug Tolerant Persisters (DTPs) that are largely quiescent and maintain viability in conditions where other cancer cells are killed (9). Drug tolerance is a temporary condition, which can revert after the cessation of cytotoxic stimuli. Differently, in the presence of continuous drug stimulation or other cellular stresses such as hypoxia, drug tolerance stabilizes into an enduring drug resistant state (9, 10). Besides quiescence, senescence has also been proposed as a process adopted by tumor cells to escape from therapy (11), suggesting that drug resistance is a composite picture of heterogeneous cell states. This picture is further complicated by a plethora of cell-intrinsic and extrinsic factors that contribute to the establishment of drug resistance including hypoxia, cytokines (among which IL-6, IL-8, and TGF-β play a prominent role), cellular composition and stiffness of the extracellular matrix. Drug resistant cells are found not only within bulk tumor populations but are also scattered in distant organs as disseminated tumor cells (DTCs), which have been recognized as the seeds of metastasis. DTCs are in a state of dormancy, which is induced and maintained by interactions with the target organ niche (12). The neutralization of DTCs is a primary goal in patients with cancers subject to late relapses such as breast and prostate cancer: in fact, recent insights on the mechanisms by which DTCs persist and reawaken are paving the way for new therapeutic avenues (13). This review will draw a picture of drug resistant cells in different contexts such as primary tumors or pre-metastatic niches and discuss a surge of recent findings that shed new light on their strengths and weaknesses, making drug resistance one of the most fertile fields of cancer research.

## Drug Resistant Cancer Stem Cells: A Concentrate of Robustness and Plasticity

The concept of CSCs originated as a hierarchical model where, in parallel to normal tissues, a small number of undifferentiated elements give rise to intermediate progenitors and finally to a differentiated progeny. While the hierarchical model remains fundamentally valid for normal tissues (with the exception of rare dedifferentiation events occurring during tissue regeneration or artificial reprogramming), it is becoming clear that boundaries between stem and non-stem cells are much weaker in cancer. In fact, in tumors state transitions seem to be very frequent and chaotic, thus generating high levels of heterogeneity that constitute the foundation of drug resistance (14–16). Not surprisingly, state transitions also affect the expression of molecules expressed on the cell membrane, such as surface markers used for CSCs isolation. Expression of surface or intracellular markers can in some cases identify a population of cells with enhanced self-renewal and/or metastatic capacity in several tumors (Supplementary Table 1). However, it should be kept in mind that such expression is transient, dynamic, and variable both among individual tumors (17, 18). In fact, few past studies on the expression of CSCs markers analyzed the expression of such markers over time (particularly upon flow cytometry isolation of positive and negative populations) or the variation of CSCs markers upon microenvironmental stimuli. Consequently, the phenotypic plasticity and dynamic properties of CSCs populations were often overlooked. Functional features such as tumor-repopulating ability in limiting dilution/serial transplantation assays are more suitable to identify CSCs populations. Such assays may nonetheless select for particularly robust cells able to thrive in harsh experimental conditions. Genetic barcoding makes use of lentiviral infection systems to tag human cells and has been employed to analyze and track stem cell hierarchies, particularly in colorectal cancer. In this context, molecular tracking studies revealed a stable functional heterogeneity of the colorectal CSCs population during serial xenografting despite profound changes in genomic subclone contribution (19), thus highlighting the functional robustness of cancer cell hierarchies. In addition to cell-intrinsic features, interactions with the tumor microenvironment are increasingly recognized as crucial determinants of stemness. The fact that soluble molecules released by the tumor microenvironment have the potential to initiate CSC-like programs was first demonstrated in brain tumors, where the self-renewal and proliferation of stem-like cells were shown to crucially depend from their interaction with endothelial cells (20). The tumor endothelium has also been shown to produce nitric oxide, which diffuses to neighboring glioma cells and activates the Notch pathway to induce stem-like characteristics (21). Later studies in colorectal cancer showed that cancer-associated fibroblasts secrete hepatocyte growth factor, osteopontin, and stromal-derived factor 1α, which activate the WNT pathway to promote cancer cell stemness (22, 23). Tumor-associated macrophages play also a role in supporting breast CSCs and brain CSCs, further supporting the importance of the niche in dictating a cancer stem cell phenotype (24, 25). Moreover, exosomes and microvesicles produced by niche cells are increasingly recognized to influence CSCs and drug resistance. For example, microvesicles produced by breast cancer-associated fibroblasts transfer miR-221 to cancer cells thus increasing the drug resistant CD133^hi^ stem cell population (26). In addition to soluble factors, other microenvironmental features such as clone location have been recently shown to determine the self-renewal capacity of colorectal cancer cells (27). In light of these evidences, stemness in cancer can be defined as a transient state of enhanced plasticity and robustness crucially influenced by microenvironmental signals, including interactions with niche elements, tumor, and non-tumoral cells, soluble factors, and anticancer therapies. The link between stemness and drug resistance derives mainly from three observations: (1) CSCs populations are more resistant to therapy (28), (2) cancers with a stemness-related gene expression have a worse prognosis (29–33), and (3) cells with combined features of stemness, drug resistance, and dormancy have been identified in several tumors including pancreatic carcinoma (34, 35), ovarian cancer (36), melanoma (37), lung cancer (38), and CML (39). More recently, dormant/slow cycling CSCs have been identified in acute leukemia (40), glioblastoma (8, 41, 42), breast (43), and colorectal cancer (44, 45). An interesting association between stemness and dormancy, together with enhanced migratory features, has also been reported in early metastatic cells which are largely responsible for tumor dissemination (46–49). While increasing evidences point to the presence of drug resistant CSCs in multiple cancers, the effect of conventional, and targeted therapies is not usually evaluated specifically on the CSCs compartment, rendering difficult to estimate CSCs permanence after therapy. Likewise, current diagnostic and therapeutic approaches include few tools for the identification, quantification, and elimination of drug resistant cells and DTCs. The elucidation of mechanisms of drug resistance and the identification of biomarkers of resistant cells are therefore essential to improve the clinical management of cancer patients.

## Heterogeneity of Therapy Resistant Cancer Stem Cells

**Figure 1:**
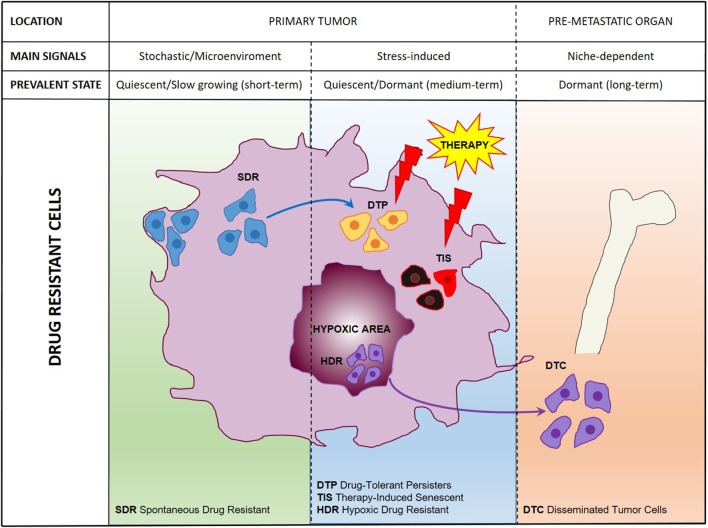
A comprehensive scheme of drug resistant cancer stem cells. Representation of main cell populations responsible for drug resistance present in the untreated tumor (SDR, Spontaneous Drug Resistant) induced by therapeutic treatment (DTPs, Drug-Tolerant Persisters; TIS, Therapy-Induced Senescent; HDR, Hypoxic Drug Resistant) or disseminated in pre-metastatic organs (DTCs, Disseminated Tumor Cells).

**Figure 2:**
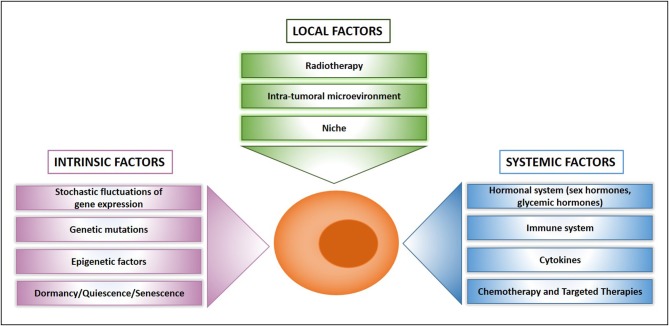
What makes a drug resistant cell. Schematic presentation of main factors that influence drug resistance at the cellular, local, and systemic level.

Previously thought to be a quite homogeneous condition, drug resistance is emerging as a surprisingly heterogeneous state that includes quiescent, drug-tolerant, and persister cells (50). This scenario is further complicated by the recent addition of post-senescent cells to drug-resistant cells responsible for disease recurrence (11). In this section we propose a functional distinction of drug resistant cells based on their origin, location, and cellular state. Figure 1 illustrates schematically three main populations associated with drug resistance such as (1) “spontaneous” drug-resistant cells in untreated tumors, (2) stress-induced drug resistant cells (including drug-tolerant persisters, post-senescent cells, and cells resident in hypoxic tumor areas), and (3) DTCs. As mentioned previously, a small population of drug-resistant cells is already present in tumors before any kind of treatment (32, 34, 37–40, 42, 44, 45). Recent studies showed that populations of endogenous drug resistant cells transiently arise as the result of stochastic state transitions that induce a high expression of resistance factors (9, 51). Such factors have been identified in melanoma cells and include EGFR, NGFR, WNT5A, AXL, PDGFRB, and JUN, with cells expressing more than one factor displaying a higher level of resistance (51). The emergence of drug tolerant cells has been detected and quantified in multiple tumors upon treatment with chemotherapeutics or targeted agents including cisplatin (in NSCLC), erlotinib, and gefitinib (NSCLC), lapatinib (breast cancer), the RAF kinase inhibitor AZ628 (melanoma and colorectal cancer), and the MET inhibitor PF-2341066 (MET-amplified gastric cancer) (9). DTPs represent a variable percentage of the parental cell population ranging approximately from 0.2 to 5% and have been identified as largely quiescent cells, although a minor part of them can resume proliferation even in the presence of the drug (9). A particularly interesting mechanism that leads to the emergence of DTPs has been recently elucidated in melanoma cells that develop resistance to BRAF inhibitors (BRAFi). A subset of melanoma cells constitutively activates the Aryl hydrocarbon Receptor (AhR), a basic helix-loop-helix transcription factor responsible for the de-differentiation of melanoma cells and the expression of BRAFi- resistance genes. Treatment with BRAFi results in the enrichment of a small subpopulation of AhR-activated BRAFi-persister cells, responsible for melanoma relapse (52). While spontaneous resistant cells and DTPs can be appropriately defined as “quiescent” as their cell cycle interruption is transient and programmed to last until the subsequent change of gene expression or drug concentration. However, such quiescent state can be stabilized by a protracted environmental stresses like hypoxia, thus shifting the balance from a short-term quiescent state to medium- or long-term dormancy. Interestingly, cells deriving from a hypoxic tumor microenvironment have been shown to activate a dormancy program giving rise to chemoresistant DTCs, further strengthening the link between dormancy and drug resistance (10). Recently, senescence has emerged as another key cellular response to drug resistance, further contributing to the heterogeneous picture of drug resistant cells. Chemotherapy and targeted therapies can induce senescence in tumor cells, intended as a stable form of growth arrest (11, 53, 54). Senescent cells are characterized by peculiar morphological features, by the expression of senescent markers (mainly SA-β-Gal and H3K9me3) and by the so-called Senescence-Associated Secretory Phenotype (11, 55). Moreover, senescent cells are arrested in the G1 or G2/M phase of the cell cycle, differently from quiescent cells that are in G0 or G0/G1 transition. Importantly, an estimated 1/10^6^ cells can escape from senescence and re-enter the cell cycle, gaining increased aggressiveness and tumor-initiating potential (56). Cells able to escape from senescence express nuclear β-catenin and stem cell markers, indicating they underwent a process of cellular reprogramming that rendered them a fully functional cancer stem cell population (56). Finally, DTCs represent a category of drug resistant stem cells located in lymph nodes or distant organs that can persist for decades after removal of the primary tumor. DTCs are crucially dependent from niche interactions and can be resistant to chemotherapy, targeted therapies, and hormonal therapy (57–59). Thus, understanding the biology of DTCs is crucial for devising alternative strategies aimed at eradicating DTCs while dormant or preventing their awakening (12, 60). Many efforts are currently dedicated to answer key unresolved questions regarding DTCs, such as which pathways are involved in maintaining DTCs dormancy, how DTCs evade immune surveillance and what triggers their awakening (12, 60, 61). However, it is also of note that physiologic models for cancer cell dissemination are represented by orthotopic/metastatic tumors in mice, which are relatively straightforward in the case of breast cancer but less feasible in other tumors. An increased use of orthotopic/metastatic models is therefore, warranted to improve the knowledge on tumor cell dissemination, dormancy, and reawakening. A provocative contribution to the field of DTCs came from recent studies showing that disseminated breast cancer cells are protected from chemotherapy through integrin-mediated interactions irrespectively from their cell cycle status (57). Disrupting the interactions between DTCs and the perivascular niche with integrin inhibitors results in DTCs chemosensitization and may represent a clinical strategy to eradicate minimal residual disease (57). In summary, it appears that both cell-intrinsic factors and cell-extrinsic signals (either local or systemic) crucially contribute to drug resistance (Figure 2). Therefore, integrated approaches able to interfere with the establishment of drug resistance at multiple levels are urgently needed to increase the life expectancy of cancer patients.

## Non-Genetic Pathways Involved in Drug Resistance

Pioneer studies on dormant cells and their microenvironment have led to a deeper understanding of drug resistance (62), thus paving the way for a flurry of recent studies in this field. Here, we will discuss the main categories of factors crucially involved in the determination of a dormant/drug resistant status with particular focus on recent discoveries. It is important to acknowledge that factors responsible for dormancy/drug resistance are not mutually exclusive, but many of them are likely active at the same time and crosstalk to reinforce each other.

### Stress-Induced Pathways Part 1: The p38 Hub

Early studies pointed to a key role for p38 stress-activated protein kinase activation in dormant cells indicating that the balance between proliferation and dormancy is determined by the ratio between the activity of p38 and ERK1/2 (63, 64). Since then, p38 has emerged as a hub in the control of multiple pathways involved in both drug resistance and cellular stress and has itself reported to induce chemoresistance in a variety of tumors. Further insights into p38-activated pathways led to the key discovery that the orphan nuclear receptor NR2F1 upon activation by p38 induces dormancy through SOX9, RARβ, CDK inhibitors, and global chromatin repression in head and neck squamous cell carcinoma (65). Recently, NR2F1 has emerged as a clinical marker of dormancy, its expression being able to discriminate breast cancer patients with short term systemic relapse from those with long disease-free intervals (66). Most interestingly, factors involved in the p38/NR2F1/retinoic acid receptors pathway are possibly emerging as part of a general program of dormancy/drug resistance active across several types of cancer. In line with this hypothesis another retinoic acid-binding nuclear receptor, RORγ, emerged during a recent mapping of molecular traits related to stemness and drug resistance in pancreatic cancer (67). RORγ, which is also known for its role in immune modulation ad inflammation, was correlated with the aggressiveness of pancreatic cancer and its inhibition led to a striking defect in tumor growth (67). A current clinical trial based on the use of 5-azacytidine/all-trans retinoic acid aimed at inhibiting DTCs reawakening in prostate cancer patients (NCT03572387) will provide clinical evidence on the efficacy of epigenetic therapies that induce histone demethylation and NR2F1 activation. SOX9 is another factor downstream of p38 that has recently been involved in drug resistance of CSCs in breast and esophageal cancer (68, 69) and in chemoresistance of cholangiocarcinoma (70). Interestingly, SOX9 expression is regulated by the SCF^FBW7^ (Skp1/Cul1/F-box), a component of the ubiquitin ligase complex (71, 72), which has been recently shown to regulate dormancy/drug resistance in breast cancer. In fact, Fbxw7 ablation sensitizes disseminated breast tumor cells to chemotherapy, arguing for a key role of the ubiquitination pathway in dictating drug resistance by selective substrate degradation (72). SOX2 and SOX9 transcription factors are also typically expressed by dormant CSCs in breast and lung cancer. Interestingly, these cells maintain dormancy in an autocrine fashion by inhibiting Wnt signals through expression of the Wnt inhibitor DKK1 (73). Other tumors have been recently reported to modulate Wnt signaling in order to maintain dormancy: prostate cancer cells receive Wnt5a from the osteoblastic niche and activate a non-canonical signaling that represses canonical Wnt3a/β-catenin signaling (74). Notably, osteoblast-produced Wnt5a acts by inducing the Siah E3 Ubiquitin Protein Ligase expression, further supporting the role of E3 ligases in dormancy/drug resistance (75). Wnt signals have also been implicated in the survival of dormant tumor cells in colorectal cancer, which have been identified as a population of partially differentiated cells characterized by high clonogenic capacity and chemoresistance (45).

### Stress-Induced Pathways Part 2: Hypoxia, Endoplasmic Reticulum Stress, and Autophagy

Global stress responses such as the hypoxia, unfolded protein response (UPR), endoplasmic reticulum stress and autophagy have all been implicated in drug resistance and pre-metastatic dormancy. Hypoxia has been habitually linked to tumor aggressiveness and poor survival but the underlying mechanisms are still under elucidation. Cells in low oxygen microenvironments activate hypoxia-inducible factors and increase the expression of key dormancy genes such as N2RF1, p27, and MIG6, inducing a combined state of dormancy and drug resistance (10, 76). Hypoxic responses can be triggered by hypoxic microenvironments in primary tumors but can also be induced by chemotherapy, which promotes a signaling cascade involving calcium release from the endoplasmic reticulum and expression of pluripotency genes, leading to an enrichment of stem cells in breast cancer (77). The endoplasmic reticulum and the related UPR, which is responsible for re-establishing endoplasmic reticulum homeostasis following cellular stress, are implicated in several steps of the drug resistance process (78). The endoplasmic reticulum stress sensor GRP78, previously shown to be downstream of activated p38 (79), seems to play a central role in the induction of drug resistance and has been particularly investigated in pancreatic cancer, where it has been involved in both chemoresistance and maintenance of the stem cell population (80, 81). Besides chemoresistance, endoplasmic reticulum stress has been demonstrated to be involved also in resistance to tyrosine kinase inhibitors in lung cancer, where drug persister cells activate the recently described ufmylation pathway and downstream UPR to upregulate key survival signals such as Bcl-xL (82). An interesting crosstalk occurs between endoplasmic reticulum stress/UPR and autophagy, which occur simultaneously and are both implicated in tumorigenesis and chemoresistance. In fact, GRP78, PERK, and ATF6 lie at the crossroads between UPR and autophagy, being able to modulate both pathways (83). Autophagy was recognized a decade ago as being implicated in the regulation of tumor cell survival and dormancy in ovarian and gastrointestinal tumors (84, 85). In fact, autophagy is required during quiescence for recycling of aminoacids and nucleotides (86), but new evidence adds to a specific role of autophagy in dictating chemoresistance in colorectal cancer (87), liver cancer (88), brain tumors (89), and melanoma (90). Recently, autophagy has been shown to be essential for the survival of disseminated dormant breast cancer cells and its inhibition with antimalarial hydroxychloroquine eliminates DTCs while dormant (91). In KRAS-dependent tumors such as pancreatic adenocarcinomas (PDAC), KRAS inhibition has demonstrated to increase autophagic signaling resulting in autophagy dependance. Removing this protective mechanism through the combined use of MEK/ERK inhibitors and autophagy inhibitors may be therapeutically beneficial in patients with PDAC, NRAS-driven melanoma, and BRAF-mutant colorectal cancer (92). By contrast, the activation of autophagic/lysosomal pathways can occur as the consequence of anticancer therapies, as has been demonstrated in melanoma treated with anti-BRAF targeted agents (93). In this case, autophagy blockade has detrimental effects, resulting in enhanced tumor progression, metastatic dissemination, and chemoresistance. Thus, autophagy may play different roles in multiple contexts and further studies are needed to clarify the potential utility of autophagy modulators in cancer therapy.

## Metabolic Reprogramming of Drug Resistant Cells

Metabolic deregulation is recognized as a hallmark of cancer, and increasing evidences suggest that it can be exploited by neoplastic cells in order to acquire a drug resistant phenotype. Intuitively, since chemotherapy kills highly proliferative cells that rely on aerobic glycolysis, it also induces a selective pressure toward the emergence of slow growing cells switched to OXPHOS metabolism (94). However, this apparently straightforward hypothesis is contradicted by very different metabolic patterns found in resistant tumor cells (95). On one hand, several studies indicate that chemotherapy-resistant cells become OXPHOS-dependent (96–99). However, other reports showed that chemoresistant cells rely on high ATP levels (100) and express glycolytic markers (101–104). A possible explanation for such divergences can be found in the timing of data collection relatively to drug treatment: during therapy, resistant cells may activate a survival program based on proliferative slowdown and switch to OXPHOS, while some time after therapy cessation cells may recover a high proliferative rate associated to aerobic glycolysis. However, the main explanation for the different metabolic patterns found in drug resistant cells probably resides in the high plasticity of the metabolic response to cytotoxic challenges. A recent study confirmed this hypothesis by showing that cancer cells are able to switch between OXPHOS and glycolysis to circumvent the inhibition of either process (105). A therapeutic strategy targeting metabolic plasticity based on intermittent fasting (to reduce glucose availability) plus the OXPHOS inhibitor metformin effectively restrained tumor growth by activating PP2A, GSK3β, and the pro-apoptotic protein Mcl-1 (105). These results also suggest that an optimization of metformin administration schedules may potentiate its ability on tumor metabolism and increase its therapeutic efficacy. Metabolic stress is a condition often encountered by tumor cells, particularly by the quiescent population resident in poorly vascularized/hypoxic areas. The combination of hypoxia and reduced nutrient availability limits the metabolic plasticity of tumor cells, which become more sensitive to drugs that target mitochondrial respiration. In fact, drugs targeting mitochondrial bioenergetics have been proposed to eliminate metabolically stressed quiescent cells, alone or in combination with autophagy inhibitors (106). Interesting insights into the mechanisms of drug-induced metabolic reprogramming have come from the study of estrogen receptor (ER)-positive breast cancers. In these cancers, hormonal therapy has been shown to result in the emergence of dormant CD133^high^/ER^low^ cells responsible for metastatic progression and to induce an OXPHOS metabolic editing of breast cancer cells through IL6/Notch3 signaling (107). Hormonal therapy resistance and the generation of breast CSCs have been correlated to microvesicle-mediated horizontal transfer of microRNAs from host stromal cells (26). Recently, extracellular microRNAs have been further implicated in the metabolic crosstalk between tumor cells and their microenvironment by showing that cancer cell-secreted miR-105 instructs cancer-associated fibroblasts to display different metabolic features, thus helping the tumor to face changes in the metabolic environment (108). Finally, metabolic reprogramming related to the development of drug resistance has been also shown to occur during antiangiogenic therapies (109). Interestingly, overexpression of the glucose transporter 3 (GLUT3) recapitulates all the metabolic features of bevacizumab-resistant cells indicating GLUT3 as a potential metabolic target in glioblastoma (110). In summary, it appears increasingly clear that metabolic heterogeneity can be driven by both intrinsic (either genetic or epigenetic) mechanisms or as an adaptation to environmental changes and plays a key role in the development of drug resistance, representing a potential avenue for targeted therapies (111).

## Epigenetic Plasticity in the Regulation of Drug Resistance

Epigenetic deregulation is a feature of virtually all human cancers (112). Tumors exhibit a continuously changing epigenetic landscape that includes altered modifications of DNA promoter regions, deregulated acetylation, or methylation of histone proteins or inappropriate expression of repetitive regions, contributing to tumors biological properties (113). The involvement of epigenetic (rather than genetic) mechanisms in drug resistance is particularly evident when drug resistant states are transient, rapidly emerging, and functionally heterogeneous. A number of past studies have demonstrated a contribution of epigenetic modifiers such as histone deacetylases (HDACs) to oncogenesis with different mechanisms strongly depending on the cellular context (114). Recent observations point to a crucial role of histone demethylases, and in particular KDM2, KDM3, KDM5, KDM6, and KDM7, in generating a drug resistant state and often a concomitant slow-dividing and stem cell-like state (8, 9, 115–121). Additional interesting insights into the epigenetic mechanisms of drug resistance came from the observation that drug treatment induces a rapid reprogramming of spontaneous resistant cells in primary tumors, converting the transient quiescent state into a stably resistant state and generating DTPs, the cells that actually survive drug-induced toxicity (Figure 1) (51). In breast cancer, multiple epigenetic enzymes including KDM5B, bromodomain, and extraterminal (BET) proteins and the histone methyltransferase EZH2 have been shown to be responsible for the generation of persister cells through a dynamic remodeling of the chromatin architecture, and such state transitions can be counteracted with inhibitors of chromatin-modifying enzymes (117). Recently, BET inhibitors were found to revert drug resistance and to block the pro-tumorigenic activity exerted by YAP/TAZ binding to the epigenetic coactivator bromodomain-containing protein 4 (BRD4) (122). However, cancer cells can develop also resistance to epigenetic inhibitors. In neuroblastoma, PI3K pathway activation and transcriptional reprogramming can confer resistance to BET inhibitors, indicating that sequential or combination therapies will likely be required to achieve durable antitumor effects (123). In this regard, the combined inhibition of BET proteins and HDACs is increasingly regarded as a strategy to improve the effectiveness of these drugs in cancer (124). A novel link between epigenetic regulation and chemoresistance has come from colorectal cancer, where the epigenetic dioxygenase TET2 has been shown to control a population of slow cycling cells responsible for chemoresistance and tumor recurrence (44). Slow cycling cell populations generated by epigenetic factors in multiple tumor settings likely represent a reservoir for the subsequent emergence of heterogeneous proliferating drug resistant cells. In fact, further epigenetic rearrangements and even genetic mutations can occur in quiescent cells giving rise to a variety of survival strategies (125). In line with this hypothesis, a single lung cancer persister cell was shown to generate a variety of colonies with different mechanisms of erlotinib resistance (126). Finally, recent discoveries suggest that repetitive transposable elements may be involved in the epigenetic determination of drug resistance. Repetitive elements constitute nearly half of the human genome and in normal cells they are tightly regulated to avoid dangerous inappropriate activation events. In cancer cells repetitive elements are often aberrantly activated, in part due to decreases in DNA methylation (127). Chemotherapeutic and targeted drugs can also induce a strong activation of repeated elements in cancer cells that results in cell death (116, 128). By contrast, DTPs within the heterogeneous cancer cell population are able to maintain the epigenetic repression of repetitive elements through increased histone H3K9 and H3K27 methylation even during drug treatment, exploiting this strategy to survive drug exposure (116). In line with these observations, a new generation of drugs targeting epigenetic modulators are finding their way to the clinic, in the attempt to exploit cancer-associated epigenetic traits for therapeutic intervention (129). Clinically induced derepression of genomic repeat elements also harbors the potential to enhance the immunogenicity of cancer cells and enhance the response to immunotherapeutic approaches (127), fostering further investigations on the mechanisms that deregulate repeat element expression in tumor cells.

## Interactions Between the Immune System and Therapy Resistant Cells

The importance of the immune system in controlling tumor growth, metastatization, and relapse is undisputed, as witnessed by the expanding role of immunotherapies in cancer treatment. New challenges concerning the use of immunotherapeutic drugs are often related to properties of CSCs, which have been reported to have a low immunogenic profile and peculiar interactions with immune cells (130). A striking example of interactions between CSCs and immune cells resulting in immunotherapy resistance has been recently highlighted in squamous cell carcinoma. Here, a population of tumor-initiating cells responsive to TGF-β acquires the expression of CD80 (a molecule previously identified on cells of the immune system) and hinders cytotoxic T cell activity leading to tumor relapse (131). The relationships between drug resistant cancer cells and the immune system are the object of particularly intense investigations in the pre-metastatic context. In fact, while tumor cells in the primary tumor are often surrounded by an immune-suppressive microenvironment (132), DTCs are theoretically more vulnerable to immune attack, providing a window for therapeutic interventions aimed at preventing metastasis formation. Recent reviews have addressed in detail the role of the immune system in cancer (133) and the mechanisms of DTCs immune escape (60, 134). Here, we will focus our discussion to a limited number of recent breakthroughs in the relationships between drug resistant cells and the immune system. New insights on how DTCs evade immune recognition came from the observation that dormant cells activate the UPR, which in turn causes the downregulation of major histocompatibility complex class I (MHC I) molecules required for antigen presentation to CD8^+^ T cells. This mechanism rendered DTCs undetectable by CD8^+^ T cells, while targeting the UPR led to MHC I re-expression and reversal of the immune-evasive phenotype (135). The most important soluble factors in cancer-immune system interactions are interferons, and particularly IFNγ produced by T cells and NK cells (136). IFNγ has been shown to induce cancer cell dormancy through multiple pathways and, interestingly, to exert different effects in indolent (Ki67^low^) cells, and in dormant (Ki67^−^) cells (137). Besides its established role in contributing to anti-tumor immunity, IFNγ is also implicated in mediating resistance to various cancer therapies, including anti-PD1 therapies via downregulation of MHC I molecules (138, 139). An interesting link between IFNγ and CSCs metabolism came from the observation that IFNγ triggers cancer dormancy through indolamine 2,3 dioxygenase (IDO1), an enzyme that catalyzes tryptophan metabolism. Blocking IDO1 metabolic circuitry abrogates dormancy and induces apoptosis of tumor-repopulating cells (140). The same metabolic pathway was found to be involved in IFNβ-induced dormancy in melanoma (141). The Stimulator of Interferon Genes (STING) is a central component of the intracellular DNA sensing pathway and has been initially characterized for its capacity to mediate type I interferon inflammatory responses in immune cells during infections. Recent breakthroughs indicate that the STING pathway has much broader functions, being implicated also in fundamental cancer-related processes such as cellular transformation (142, 143), metastasis (144), and response to radio- and chemotherapy (145, 146). STING has been recently identified as an activator of autophagy downstream of the ancestral cyclic GMP-AMP synthase (cGAS) pathway (147) and may also be implicated in chemoresistance-related autophagy. Additional evidences indicate a direct link between STING and LKB1, which is a crucial regulator of stem cell quiescence, metabolism, and anti-tumor immunity (148). Specifically, loss of LKB1 leads to the suppression of STING and insensitivity to cytoplasmic double-strand DNA detection, resulting in resistance of lung cancer to immunotherapy (149). Therefore, therapies that reactivate LKB1 or the STING pathway may boost anticancer immune response in cancers with resistance to immune-checkpoint blockade (150). Finally, immune cells have been recently implicated in DTC reawakening from dormancy in a study showing a key role for Neutrophil Extracellular Traps (NETs) produced by neutrophils in the lung parenchyma upon inflammation. NETs trigger integrin-mediated activation of focal-adesion kinase in DTCs and subsequent exit from dormancy, while integrin-blocking antibodies prevent DTC reactivation in NET-enriched lungs (151). The latter study confirms the involvement of integrins in chemo- and radiotherapy resistance of multiple cancers, raising hopes for the future development of effective therapeutic agents blocking integrin signaling (152).

## Considerations on Targeting Therapy Resistant Cancer Stem Cells

Therapies directed against drug resistant cells resident in either primary tumors, pre-metastatic niches, or metastatic cancers have to face an array of genetic and epigenetic survival strategies exploited by cancer cells. Conventional therapies such as radio- and chemotherapy, although representing the mainstay of cancer therapy, are intrinsically limited in their capacity to face drug resistance and may themselves promote the emergence of more aggressive cells. In recent years the mechanisms underlying cancer cell plasticity, heterogeneity, stress responses, and dormancy have begun to be elucidated, indicating new routes of therapeutic intervention. At the same time, new therapeutic approaches should undergo careful pre-clinical evaluation for their effects on the CSCs compartment, which would provide indications on the development of resistant cell populations. New therapeutic strategies directed against dormant/drug resistant cells in primary tumors are progressively focusing on epigenetic modulators such as inhibitors of KDMs, HDACs, or BET proteins. By contrast, therapeutic strategies directed against pre-metastatic DTCs are divided in three main workstreams: the so-called “sleeping strategies” include drugs that suppress proliferative signals such as anti-estrogen therapies (153), inhibitors of CDK4/6 (154), and inhibitors of ERK or Src (155). Prolonging dormant states can also be obtained with drugs that increase the expression of dormancy factors such as p38, DYRK1A, and N2RF1 (63, 65, 156, 157). Sleeping strategies such as anti-estrogen therapies for breast and prostate cancer had a profound impact in the clinical setting. However, sleeping strategies must be long-lasting (or even lifetime long) and therefore must deal with unwanted side effects that can limit their long-term usage and reduce patient compliance. In this regard, the use of retinoic acid or fenretinide derivatives to induce cancer cell quiescence appear as a feasible and relatively non-toxic therapeutic approach (12, 158). An additional problem related to sleeping strategies is that not all tumor cells are responsive, as ER-positive breast tumors can give rise to metastases even during hormonal therapy. Thus, the combination or sequential administration of “sleeping” therapeutics should be explored, with the caution of avoiding toxic side effects. A second type of strategies directed against dormant cells is represented by cell cycle reactivation, classically with G-CSF or IFNα (159). However, treatment with reactivating agents may not be effective on all the tumor cells, leaving behind some dormant persisters. Also, therapeutic reactivation may render tumor cells more aggressive and potentially resistant to subsequent chemotherapy. The third strategy consists in eliminating dormant/drug resistant cells while dormant, a difficult but not impossible challenge. Elimination of dormant cells has been achieved in experimental brain tumors with mithramycin (160), HDACs or KDMs inhibition (8, 9, 116, 119), while in pancreatic tumors resistant cells were eliminated by IGF1 inhibition (161). Activators of ferroptosis, a form of cell death characterized by the accumulation of lipid peroxidation products and lethal reactive oxygen species derived from iron metabolism, were shown to kill drug tolerant cells in multiple tumors (162, 163). Mitochondrial respiration is also considered a promising target (106, 164–167), although metabolic plasticity could result in the unresponsiveness of some resistant cells. Besides the inhibition of specific cellular proteins/pathways, other strategies with a broader mechanism of action may be useful to cope with drug resistance. First, immunotherapy alone or in combination with targeted agents is proving effective in several cancers even in the metastatic setting (168, 169). Adoptive transfer of tumor-reactive lymphocytes has led to striking anti-tumor immune responses in breast cancer and other tumors (170–174), indicating that totally drug resistant cells such as those in advanced metastatic tumors can still be eliminated by the immune system. Also, emerging evidences suggest that micro- and macro-environmental signals can profoundly impact on the biology of drug resistant cells. While inflammation has been shown to facilitate metastatic outgrowth (151, 175, 176), anti-inflammatory agents such as NSAIDs seem to dramatically decrease the risk of metastatic relapse, possibly by preventing the reawakening of dormant cells caused by niche alterations that occur during inflammation (177, 178). Finally, therapeutic strategies that include lifestyle-related factors such as exercise and nutrition are emerging as an important tool not only in cancer prevention but also in managing established cancers (179). Diet and lifestyle likely act through reinforcing the immune system, modulating hormone levels, shaping gut microbiota, preventing inflammatory conditions, and influencing the pre-metastatic niche to become less favorable to DTCs awakening (179, 180). A link between diet-related factors and therapy has recently emerged by studies showing that a hypoglycemic diet improves the effectiveness of PI3K inhibitors (181). Likewise, fasting or fasting-mimicking diets are increasingly considered as valid supports in cancer therapy due to their ability to induce wide alterations in growth factors and metabolite levels that generate unfavorable environments for cancer cells and improve the effects of cancer therapies (182). Finally, physical exercise is being explored for its ability to promote and restore antitumor immunity. In fact, the infiltration and antitumor activity of immune cells are limited by a scarcely oxygenated and acidic tumor microenvironment. Exercise has been reported to modulate oxygen concentration and pH in the tumor bed and to directly stimulate tumor cell killing by immune cells, thus appearing as a potential tool to improve the effectiveness of immunotherapy (183). In summary, drug resistance increasingly appears as a multifactorial process (Figure 2) where each class of factors can be considered as a target for novel therapeutic strategies.

## Conclusions

Understanding the mechanisms of drug resistance is mandatory in order to improve the effectiveness of cancer therapies. While novel and unexpected mechanisms of drug resistance continue to emerge, translational research is moving toward new therapeutic approaches involving not only cancer cells and peritumoral cells but also other components of the body such as the immune and the hormonal system. As a result of the discoveries made in the last decade, drug resistant cancer cells in their different contexts are starting to appear as a treatable target. However, increasing efforts are required to explore the mechanisms that regulate drug resistance of cancer cells either in primary tumors, pre-metastatic niches, and overt metastases in order to find new therapeutic avenues.

## Author Contributions

AZ, MD, and FF wrote the manuscript. FL provided essential expertise.

### Conflict of Interest Statement

The authors declare that the research was conducted in the absence of any commercial or financial relationships that could be construed as a potential conflict of interest.

## Abbreviations

SA-β-Gal: senescence- associated-β-galactosidase
H3K9me3: trimethylated lysine 9 at histone H3
CML: chronic myeloid leukemia
p38 MAPK: p38 mitogen-activated protein kinase
ERK 1/2: extracellular signal-regulated kinase ½
RARβ: retinoic acid receptor beta
RORγ: nuclear hormone receptor retinoic acid receptor-related orphan receptor gamma
SOX9 (sex-determining region Y [SRY]–containing box 9)
GRP78: glucose regulatory protein 78
EZH2: enhancer of Zeste homolog 2
OXPHOS: oxidative phosphorylation
NSAIDs: non-steroidal antinflammatory drugs
IGF1: insulin growth factor 1
EGFR: epidermal growth factor receptor
NGFR: nerve growth factor receptor
NSCLC: non-small cell lung cancer.
