# Supplementary material for: Stem Cell Plasticity and Dormancy in the Development of Cancer Therapy Resistance
Source: Front Oncol. 2019 Jul 10;9:626. doi: 10.3389/fonc.2019.00626 (PMC6636659; doi:10.3389/fonc.2019.00626)
Supplement: Supplementary file 1 [file Table_1.pdf]

CancerType	Stem Cell Marker	Reference (doi)
**Acute MyeloidLeukemia**	$CD34^{+}/CD38^{-}$	10.1038/nm0797-730
**Breast Carcinoma**	$ESA^{+}/CD44^{+}/CD24^{-/low}$	10.1073/pnas.0530291100
	$ALDH^{+}$	10.1016/j.stem.2007.08.014
	$ABCG2^{+}$	10.1200/jco.2013.31.15
**Colorectal Carcinoma**	$CD133^{+}$	10.1038/nature05372
	$CD44v6^{+}$	10.1016/j.stem.2014.01.009
	$EphB2^{+}$	10.1016/j.stem.2011.02.020
	$CD44^{+}/EpCAM^{hi}$	10.1073/pnas.0703478104
	$CD166^{+}$	10.1073/pnas.0703478104
	$Cripto^{+}$	10.1038/cdd.2015.19
	$CXCR4^{+}$	10.18632/oncotarget.13214
	$ALDH1^{+}$	10.1158/0008-5472.CAN-08-4418
**Lung Carcinoma**	$CD133^{+}$	10.1038/sj.cdd.4402283
	$ABCG2^{+}$	10.1158/0008-5472.CAN-06-3557
	$ALDH^{+}$	10.1158/1541-7786.MCR-08-0393
	$CD44^{+}$	10.1371/journal.pone.0014062
	$CD44^{hi}/CD90^{+}$	10.1371/journal.pone.0057020
	$uPAR^{+}$	10.1371/journal.pone.0000243
	$CD117^{+}$	10.1371/journal.pone.0003077.
	$CD166^{+}/CD44^{+}$	10.1186/s12885-015-1086-3
	$CD166^{+}/EpCAM^{+}$	10.1186/s12885-015-1086-3
**Ovarian Carcinoma**	$CD133^{+}$	10.1634/stemcells.2008-0868
	$CD44^{+}/CD117^{+}$	10.1158/0008-5472.CAN-08-0364
**Melanoma***	$ABCB5^{+}$	10.1038/nature06489
	$CD133^{+}$	10.1016/j.ejca.2007.01.017
	$CD271^{+}$	10.1038/nature09161
	$CD20^{+}$	10.1158/0008-5472.CAN-05-1343
**CNS (Glioma, Medulloblastoma)**	$CD133^{+}$	10.1038/nature03128
	$Integrin\alpha7^{+}$	10.1016/j.stem.2017.04.009
	$CD90^{+}$	10.1074/mcp.M111.010744
	$Integrin\alpha6^{+}$	10.1016/j.stem.2010.02.018
**Multiple Myeloma****	$CD138^{-}$	10.1182/blood-2003-09-3064
	$CD138^{+}$	10.1182/blood-2010-02-267344
**Prostate Carcinoma**	$CD44^{+}/\alpha2\beta1^{hi}/CD133^{+}$	10.1158/0008-5472.CAN-05-2018

**Head and Neck Carcinoma**	CD44$^+$	10.1073/pnas.0610117104
**Gastric Carcinoma**	CD44$^+$	10.1002/stem.30
**Pancreatic Carcinoma**	CD133$^+$	10.1016/j.stem.2007.06.002
	CD44$^+$/CD24$^+$/ESA$^+$	10.1158/0008-5472.CAN-06-2030
	CD133$^+$/CXCR4$^+$	10.1016/j.stem.2007.06.002
	ALDH$^+$	10.1371/journal.pone.0020636
**Hepatocellular Carcinoma**	CD133$^+$	10.1053/j.gastro.2007.04.025
	CD44$^+$/CD90$^+$	10.1016/j.ccr.2008.01.013
	ALDH$^+$	10.1158/1541-7786.MCR-08-0035
	ABCG2$^+$	10.1007/s00432-008-0407-1
**Bladder Carcinoma**	Lin$^-$ /CD44$^+$/CK5$^+$/CK20$^-$	10.1073/pnas.0906549106
	ALDH1A1$^+$	10.1158/1055-9965.epi-09-0865

Supplementary Table 1. Markers used to isolate stem-like cells in the indicated cancer types.
* The existence of a clearly defined CSCs population in melanoma was challenged in the study by Quintana et al. (Science 2010, doi: 10.1016/j.ccr.2010.10.012), who investigated 22 putative melanoma markers including CD271 and ABCB5 and showed that none of them enriched tumorigenic cells. **Two studies reporting opposite findings on CD138 as a marker for myeloma CSCs likely represent an example of dynamic marker expression.
